# Gr-1+ Cells Other Than Ly6G+ Neutrophils Limit Virus Replication and Promote Myocardial Inflammation and Fibrosis Following Coxsackievirus B3 Infection of Mice

**DOI:** 10.3389/fcimb.2018.00157

**Published:** 2018-05-15

**Authors:** Dan Xu, Peijie Wang, Jie Yang, Qian Qian, Min Li, Lin Wei, Wei Xu

**Affiliations:** Jiangsu Provincial Key Laboratory of Infection and Immunity, Institutes of Biology and Medical Sciences, Soochow University, Suzhou, China

**Keywords:** viral myocarditis, Coxsackievirus B3, neutrophil, monocyte, fibrosis

## Abstract

Coxsackievirus B3 (CVB3) is the primary cause of viral myocarditis. An early and abundant neutrophil accumulation in the myocardium is a hallmark of early CVB3 infection. Yet the relative contribution of neutrophils to host susceptibility to CVB3 myocarditis remains largely unknown. Herein, peripheral neutrophil depletion was implemented in a BALB/c mouse model of acute CVB3 myocarditis using the specific 1A-8 (anti-Ly6G) or a RB6-8C5 (anti-Gr-1) mAb covering a wide range. Anti-Ly6G treatment led to systemic neutropenia throughout the disease, but did not alter virus replication, disease susceptibility and histopathological changes in the heart and pancreas of mice. In contrast, depletion of both neutrophils and monocytes/macrophages by anti-Gr-1 mAb prior to and after infection significantly promoted susceptibility of mice to CVB3 infection which was associated with exacerbated cardiac and pancreatic viral load. However, depletion of Gr1+ cells significantly suppressed acute myocarditis and pancreatic acini destruction at day 7 post infection via reducing Ly6C^high^ monocyte population in the circulation. Additionally, cardiac interstitial fibrosis was not affected by neutrophil depletion, whereas Gr-1+ cells other than neutrophils increased cardiac fibrosis at day 21 p.i. by increasing cardiac expression of profibrotic cytokine TNF-α and TGF-β. Thus, Neutrophil function is most likely not essential for CVB3 control and peripheral neutrophils play dispensable role in the pathogenesis of acute myocarditis and pancreatitis during CVB3 infection. Whereas Gr-1+ cells other than neutrophils play a major role in limiting viral replication while promoting myocardial and pancreatic inflammatory injury and fibrosis.

## Introduction

Viral myocarditis (VMC), an inflammatory condition of the myocardium associated with several viral infections, is a significant cause of sudden cardiac death (SCD) and an underlying cause of dilated cardiomyopathy (DCM) in young adults (Sagar et al., 2012; Biesbroek et al., 2015; Pollack et al., 2015). Evidence of enteroviruses infection is found up to 25% of viral myocarditis cases (Gaaloul et al., 2014) and 50% of cardiomyopathy cases (Harris and Coyne, 2014). Coxsackievirus B3 (CVB3), a non-enveloped, single stranded RNA virus belonging to the Picornaviridae family, is still considered the dominant etiological agent causing viral myocarditis (Garmaroudi et al., 2015). CVB3-induced myocarditis is caused by initial direct viral injury and a host immune-mediated inflammatory immune injury (Fung et al., 2016). There is still no licensed vaccine or effective therapeutic treatment for human CVB3 myocarditis and cardiomyopathy (Li et al., 2015; Rose, 2016).

During the early viremia phase when cardiac viral replication peaks on 3 day post infection (dpi), innate immune responses are crucial determinants of the severity of myocardial damage, and contribute to the development of chronic myocarditis and dilated cardiomyopathy. The primary cardiac infiltrates consist of NK, γδT, macrophages, mast cell, and dendritic cells (Yajima, 2011; Corsten et al., 2012), each population may influence the local inflammatory condition and disease outcome by direct defending mechanism or by modulating of cardiac adaptive immune patterns (Massilamany et al., 2014). In murine model of CVB3 viral myocarditis, we and other groups observe an early and abundant mobilization and influx of neutrophils into the heart and the pancreas of mice following CVB3 infection as early as 2.5 dpi (Smilde et al., 2016). This mobilization is earlier than any other infiltrated innate cells. Up to 35% of the cells present in the hearts are neutrophils (Fairweather et al., 2005). And severity of acute myocarditis is strongly associated with neutrophil accumulation in the heart (Afanasyeva et al., 2004). However, there is currently no report about the role of neutrophils in experimental CVB3 myocarditis and pancreatitis.

Neutrophils play critical roles in host defense to bacterial and fungi infection (Kruger et al., 2015). They are differentiated in the bone marrow (BM) and then released into systemic circulation (Hong, 2017). Upon infection, circulating neutrophils quickly initiate chemotactic interstitial migration reaching site of inflammation (Kolaczkowska and Kubes, 2013), and protect against microbial infections through multifaceted killing mechanism (ROS, myeloperoxidase/MPO, neutrophil extracellular traps/NETs). They also crosstalk with recruited and resident immune cells, modulating immune response through the release of cytokines and chemokines (Yin and Heit, 2017). The requirement for neutrophils in host defense to bacterial and fungal infections is well described (Huppler et al., 2014). However, role of neutrophils in host defense to viral infections remains poorly characterized (Cortjens et al., 2016). Abundant neutrophil infiltration (up to 80% of infiltrated leucocytes) has been detected in the airways and lungs during human respiratory syncytial virus (RSV) (Geerdink et al., 2015) and herpes simplex virus (HSV) infections (Stock et al., 2014). Neutrophil's activities are sometimes beneficial. Neutrophil numbers increase in the respiratory tract and are among the first responders to influenza A virus (IAV) infection. They limit influenza virus replication and ameliorate lung injury from severe IAV infection (Tate et al., 2009). Neutrophils guide and enhance specific CD8+ T cells responses against influenza virus in the lung (Lim et al., 2015). However, unbalanced inflammatory neutrophil response may worsen the influenza disease outcome (Camp and Jonsson, 2017). Neutrophils also play a critical role in controlling human metapneumovirus (HMPV)-induced inflammatory responses by regulating γδ T cell lung infiltration (Cheemarla et al., 2017). In HSV infection, uncontrolled apoptosis-resistant neutrophil expansion and invasion into the brainstem in IFN-γ-deficient mice contribute to fatal viral encephalitis (Ramakrishna and Cantin, 2018). During HIV infection, increased neutrophil infiltration within the gut mucosa worsens the inflammatory condition leading to permanent damage to the gut epithelial (Yaseen et al., 2018). Since MPO and MPO-derived oxidants promote inflammation and cause tissue damage (Strzepa et al., 2017), the neutrophil to lymphocyte ratio (NLR) has become a good predictor of poor prognosis and mortality of many cardiovascular diseases (Afari and Bhat, 2016). And the neutrophil counts are strongly and independently associated with death and heart failure after myocardial infarction (Arruda-Olson et al., 2009). S100a8/a9 complex is primarily produced by infiltrating CD11b+Gr1+ neutrophils in the heart, and plays a pathogenic role in CVB3-induced myocarditis (Müller et al., 2017). As such, whether neutrophil play an antiviral role or exert detrimental effect on disease outcome in CVB3 disease is worth studying.

This study is undertaken to determine the effect of peripheral neutropenia on the pathogenesis of CVB3-induce viral myocarditis and pancreatitis in mice. We used the rat anti-Gr1 (RB6-8C5, RB6) antibody (Ab) and neutrophil-specific anti-Ly6G (IA-8) Ab to induce peripheral neutropenia in BALB/c mice and determined their susceptibility to intraperitoneal (i.p.) infection with CVB3. Our results show that RB6- but not IA-8-treated mice have exacerbated organ viral burden and mortality, but much less severe histopathology in hearts and pancreas than isotype control mice.

## Materials and methods

### Mice and virus

Six to eight week-old male BALB/c mice were purchased from Slac Laboratory Animal (Shanghai, China) and housed under pathogen-free conditions at the Soochow University laboratory animal center. Animal experiments were performed in accordance with the Institutional Animal Care and Use Committee of Soochow University. All research protocols were approved by the Animal Ethical Committee of Soochow University (SYXK2015-0036). CVB3 (Nancy strain) is a gift from Professor YingzhenYang (Key Laboratory of Viral Heart Diseases, Zhongshan Hospital, Fudan University), and was propagated in Hela cells and purified by centrifugation. CVB3 was titrated by TCID_50_ assay on Hela cell monolayers according to the Reed and Muench method.

### CVB3 infection

BALB/c mice were inoculated intraperitoneally (i.p.) with 1500 TCID_50_ of CVB3, diluted in 0.1 ml of sterile phosphate-buffered saline (PBS) on day 0. Individual experiments were conducted at least three times with 7–10 mice per group.

### Virus titers

Hearts and pancreas were aseptically removed from mice, weighed, homogenized in RPMI 1640 medium containing 5% FCS. Cellular debris was removed by centrifugation at 300 g for 10 min. Supernatants were diluted serially using 10-fold dilutions and titered on Hela cell monolayers by the TCID_50_ assay.

### Histopathology

Mice were evaluated for the development of acute viral myocarditis on day 7 or cardiac fibrosis at day 21 after infection. Hearts and pancreas were excised, rinsed in PBS and fixed in 10% phosphate-buffered formalin at RT overnight. Tissues were paraffin embedded, and serial 5 μm thick sections were cut and stained with hematoxylin and eosin or sirius red. Severity of myocarditis (marked with immune infiltration) and fibrosis (marked with collagen deposition) was evaluated from five sections per heart in a blinded manner by using a 1 to 5 scoring system (Cihakova et al., 2008): grade 0, no inflammation; grade 1 < 10% of the heart section is involved; grade 2, 10–30%; grade 3, 30–50%; grade 4, 50–90%; grade 5, more than 90%. The severity of pancreatitis (marked with acinar cell destruction) was evaluated by use of a preexisting scoring system including acinar cell necrosis and leukocyte infiltrate on a scale of 0 (absent) to 4 (extensive) as previously described (Merza et al., 2014).

### Antibody-mediated neutrophil depletion

To deplete neutrophils in mice, 250 μg (12.5 mg/Kg) of anti-Ly-6G (clone 1A8, Biolegend) and isotype control (rat IgG2a, Biolegend) Abs, or 200 μg (10 mg/Kg) of Anti-Gr-1 (clone RB6-8C5, Biolegend) in 100 μl PBS were injected i.p. into mice 24 h prior to and 3 days after inoculation with CVB3 (Day 0). To evaluate influence of neutrophils on the development of cardiac fibrosis, mice received Abs injection on days −1, 3, 6, 9, 12, 15, and 18. Blood was collected by saphenous venous puncture. Total white blood cell counts (WBC) were determined by visual enumeration after trypan blue exclusion. The percentage of neutrophils was determined by flow cytometry using antibody cocktails (anti-CD45-APC/Cy7, anti-CD11b-FITC, anti-Gr-1-PercP/Cy5.5, and anti-Ly6G-PE/Cy7, BD PharMingen). Fluorescent intensity was determined using a FACS CantoII flow cytometer (BD Bioscience) and the data were analyzed using FlowJo v10.0 software (Tree Star).

### Preparation of cardiac, pancreatic, and blood mononuclear cells

Fresh hearts were rinsed and minced into small pieces in sterile petri dishes containing 10 ml of digestion buffer which consisted of 800 μg/ml type II collagenase (Sigma), and 5 μg/ml hyaluronidase (Roche) in RPMI1640 medium plus 10% fetal calf serum (FCS). Tissues were subjected to two rounds of digestion at 37°C while stirring for 1.0 h. Then the minced hearts were passed through 70-μm mesh strainers (BD Falcon). Individual cell suspensions from three mice were pooled and centrifuged for collection of cell precipitation which was re-suspended in 40% percoll (GE Healthcare). Single cell leukocytes were isolated by 40–70% percoll followed by centrifugation for 30 min at 800 × g and re-suspended in 1640–10% FCS. Preparation procedure for pancreatic mononuclear cells was very similar to that of cardiac immune cells except the digestion buffer consisted of 500 μg/ml type IV collagenase (Sigma) and 30 μg/ml DNase I (Sigma) and acinar cells were excluded by centrifugation (50 × g, 30 s). After red blood cells were lysed by ACK buffer (eBioscience), PBMCs were isolated by centrifugation (400 × g, 10 min) of whole peripheral blood on a density gradient in Lymphocyte separation media (TBDSCIENCE, China) and re-suspended in 1640-10% FCS.

### Flow cytometry analysis of blood and heart cell populations

All antibodies were purchased from BD PharMingen (San Diego CA): CD45.2-APC/Cy7 (clone 104), CD11b-FITC (clone M1/70), Gr-1-PerCP-Cy5.5 (clone RB6-8C5), Ly6G-PerCP/Cy5.5 (clone 1A8), Ly6C-PE/Cy7 (clone AL-21), F4/80 (APC; clone BM8), CD3-PerCP (clone 145-2C11), CD4-FITC (clone RM4-5), CD8-PE (clone 53-6.7). Isotype control Abs and unstained samples were included in the analyses. Cells were incubated (20 min at 4°C) in FACS buffer (PBS, 2% FCS, 2 mM EDTA) containing an anti-mouse Fc receptor blocking reagent (Miltenyi). Afterward, cells were stained with fluorochrome-conjugated antibodies against CD45, CD11b, Ly6G, Ly6C, F4/80, CD3, CD4, and CD8 for 30 min at 4°C. Cells were acquired either on a CantoII or a FACSCalibur (both BD Bioscience). Data were analyzed using FlowJo v10.0 software (Tree Star). Reported numbers were normalized for the weight of total hearts or 1 ml blood, yielding the number of respective cell fraction per mg tissue or per ml blood.

### RNA extraction and real-time reverse transcriptase-PCR

Frozen hearts was homogenized in RLT lysis buffer (RNAeasy Kit, Qiagen, Valencia, CA). RNA was extracted with RNAeasy Kit. In all, 1 μg total RNA was used to synthesize cDNA with a SuperScript III First-Strand Synthesis System (Invitrogen). Relative quantification of indicated chemokine genes was determined by real-time PCR with SYBR Green (Quanta BioSciences, Gaithersburg, MD) normalized to GAPDH.

### Cytokine measurement

Hearts and pancreas were homogenized in PBS plus 2% FCS and centrifuged at 1,800 × g for 20 min. The supernatants were extracted and immediately frozen at −80°C, which were spun at 3,000 × g for 20 min to remove any further cellular debris before used. Blood serum were obtained by centrifugation and stored at −80°C. Cytokines (IFN-γ, IL-1β, TNF-α, IL-6, IL-10, IL-17A, TGF-β) levels in blood, heart and pancreas were measured using a commercially available enzyme-linked immunosorbent assay (ELISA) according to the manufacturer's instructions (eBioscience, San Diego, CA).

### Statistical analysis

All experiments were repeated at least three times. Data are expressed as mean ± SEM. Comparisons between groups were performed using one-way ANOVA with Bonferroni multiple comparison test. Normally distributed data on continuous parametric axes were analyzed with the Student's *t*-test. Log-rank test was used to compare survival curves. Statistical analyses were performed using GraphPad Prism 5 software. The threshold for significance was 0.05 or better.

## Results

### Abundant neutrophils are recruited into the hearts and pancreas of mice during CVB3 infection

**Figure 1:**
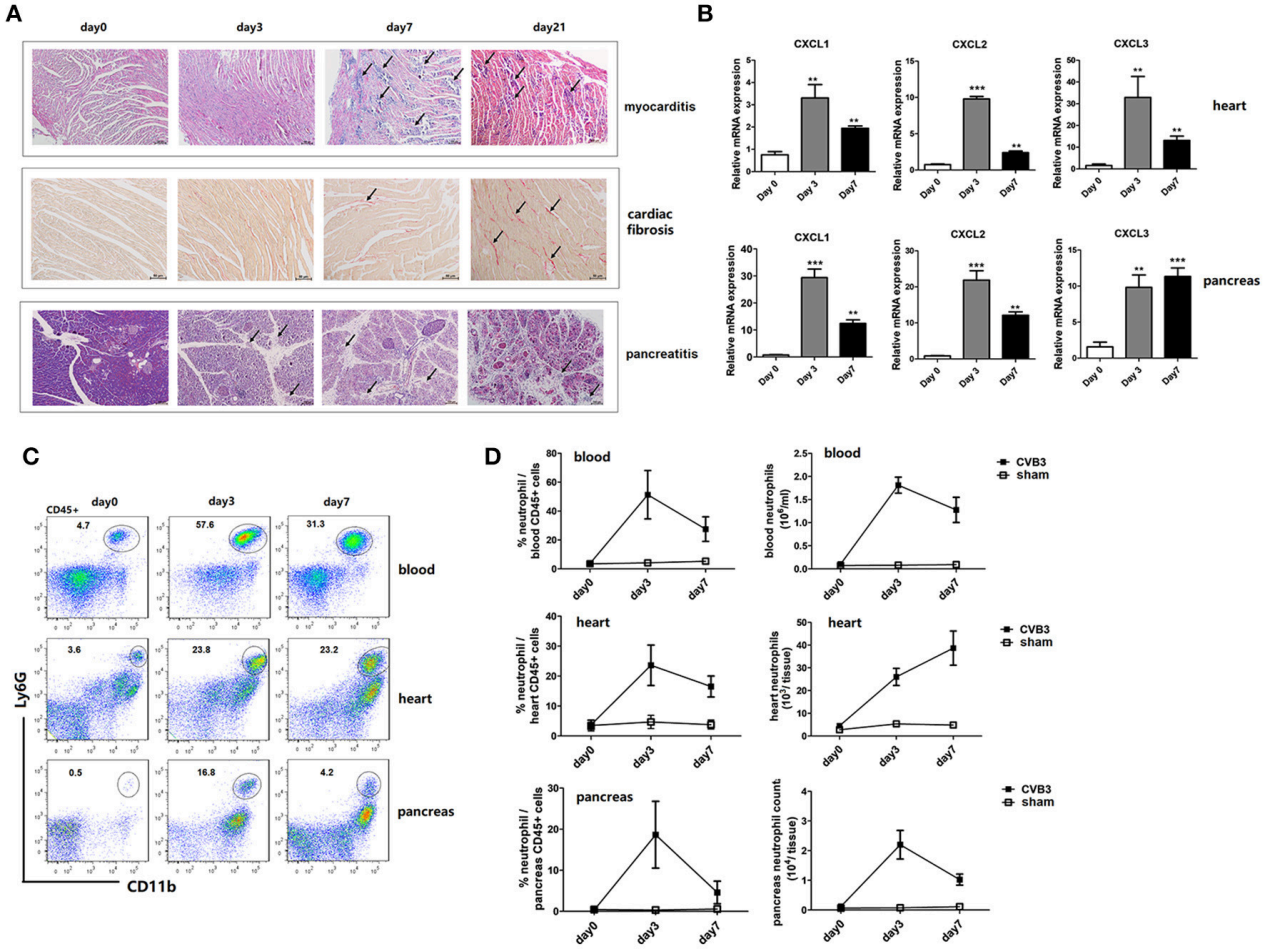
CVB3 infection induce an abundant recruitment of neutrophils into the heart and pancreas of mice. BALB/c mice were infected i.p. with 1500TCID_50_ CVB3 (5 mice/group). **(A)** H & E staining of the heart and pancreas sections showed the acute inflammatory myocarditis (arrows showing intra-cardiac immune infiltrates) and pancreatic destruction (arrows showing acinar cell lysis) during 0–7 days. Heart sections were also stained with sirius red to show cardiac fibrosis (arrows showing collagen deposition as positive for sirius red staining) during 0–21 days after infection. Scale bar: 100 μm (myocardis and fibrosis) and 50 μm (pancreas). **(B)** 0, 3, and 7 dpi, neutrophil chemokine expression in the heart or pancreas homogenates was measured by qPCR. **(C)** Representative flow cytometry plots of cardiac, pancreatic, and blood mononuclear cells stained with CD11b and Ly6G were shown. **(D)** Proportions and numbers of neutrophils were numerated in hearts, pancreas and blood of uninfected, d3 and d7 CVB3-infected mice. Results are presented as mean ± SEM, **p < 0.01; ***p < 0.001.

After inoculation i.p. with 1500 TCID_50_ CVB3, male BALB/c mice developed an acute myocarditis as well as pancreatitis at day7 post infection (p.i.). Marked diffuse mononuclear cell inflammation and massive necrosis were observed in the hearts of mice. While massive mononuclear infiltration and acinar cell destruction were seen in pancreas of mice (Figure 1A). From day 7 p.i., the inflammatory cell infiltrates decreased while myocardial fibrosis increased continuously. By day 21 p.i., massive fibrosis was observed in hearts as stained positive for collagen deposition by sirius red. During this disease, CVB3 also induced significantly up-regulated gene expression of neutrophil chemokines (CXCL1, CXCL2, and CXCL3) in both hearts and pancreas of mice compared with that in non-infected mice (Figure 1B), which peaked on day 3 and decreased by day 7 p.i. To see whether neutrophils are recruited to inflamed tissues of mice by these chemokines, frequency of neutrophils in blood and tissues of CVB3-infected mice were detected by Flow cytometry. Increased numbers of circulating and cardiac neutrophils, identified as CD45+CD11b+Ly6G+, are detectable within 24 h after CVB3 infection and continued to increase in frequency up to 2.5~3 days (55 and 23.6% in circulating and cardiac CD45+ cells, respectively) after infection (Figure 1C). And neutrophils represented the most abundant immune cells in the heart at day 3 p.i. (2.6 × 10^4^/heart, Figure 1D), with frequency and absolute numbers higher than those of other myeloid cells (macrophages and Ly6C^high^ monocytes). In the pancreas, neutrophils increased their percentage and numbers post infection, peaking and maintained high levels on day 3 p.i. then their counts decreased at 7 dpi. Our data indicate that an influx of neutrophils into the hearts and pancreas of mice is induced by CVB3 infection.

### 1A8 and RB6 mAbs efficiently deplete circulating neutrophils

**Figure 2:**
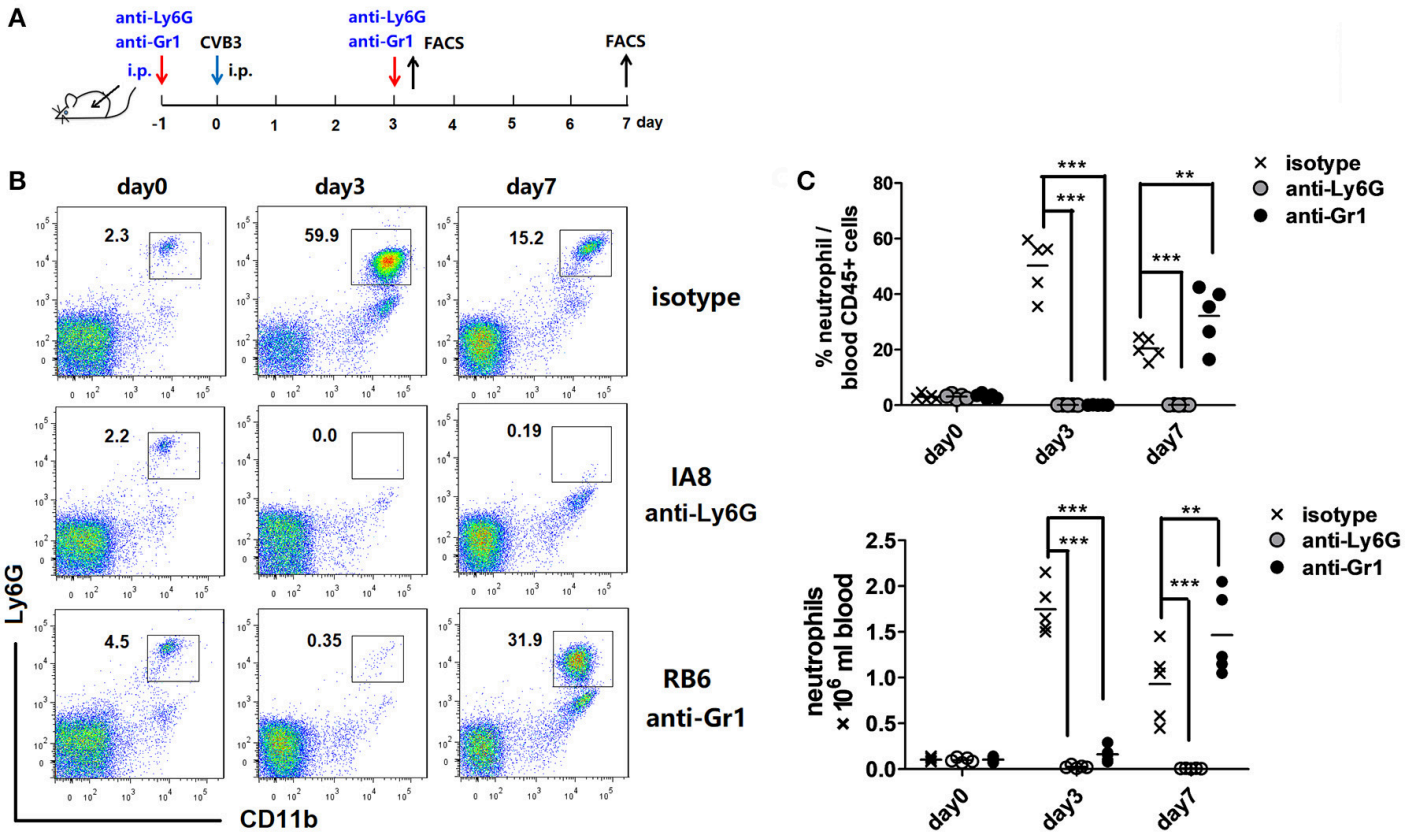
Neutrophil depletion efficiency. **(A)** Study design. BALB/c mice were treated i.p. with isotype matched IgG, anti-Ly6G(1A8, 12.5 mg/Kg) or anti-Gr-1(RB6-8C5, 10 mg/Kg) Abs on day −1 and 3, and subjected to CVB3 i.p. infection on day 0. Peripheral neutrophil counts were enumerated by flow cytometry. **(B)** Representative flow cytometry dot plots of CD45+ cells in peripheral blood leukocytes at 3 and 7 dpi. Time course depicting proportion of CD11b +Ly6G+ neutrophils against CD45+population in the blood from 0 to 7 dpi. **(C)** Time course depicting total numbers of Ly6G+ neutrophils in blood from 0 to 7 dpi. Data pooled from three independent experiments, *n* ≥6 mice per group. Results are presented as mean ± SEM. **p < 0.01; ***p < 0.001.

In order to characterize the roles of neutrophils during CVB3 infection, we used two kinds of Abs to deplete peripheral neutrophils. Anti-Ly6G (1A-8) is specific while anti-Gr-1 (RB6) depletes both neutrophils and inflammatory monocytes. One injection of 1A-8 or RB6 Ab led to a neutropenia state which persisted for 4~5 days (data not shown). Thus we developed a 2 injection experiment strategy to deplete circuiting neutrophils. To test the effectiveness of this strategy, Abs were injected into mice 1 day prior to and 3 days after CVB3 infection (Figure 2A). The circulating neutrophils were monitored every 3 days by flow cytometry. The accumulation of neutrophils in blood were completely blocked by the 2 Abs on 3 dpi (percentage as 0.1 and 0.1% in CD45+ cells, Figures 2B,C). However, by 7 dpi, anti-Ly6G mAb still reduced 96% of neutrophil counts; while a great amplification of CD11b+ Ly6G+ neutrophils were observed upon anti-Gr1 treatment (32.2% vs. 20.4% in CD45+ cells, anti-Gr1 vs. isotype, *p* < 0.01). This likely reflects an increased Bone Marrow expansion of myeloid cells resulting from high viral burden, not a defect in neutrophil depletion.

### Neutrophil depletion does not alter CVB3 infection in mice while anti-Gr1 treatment dramatically increases mortality

**Figure 3:**
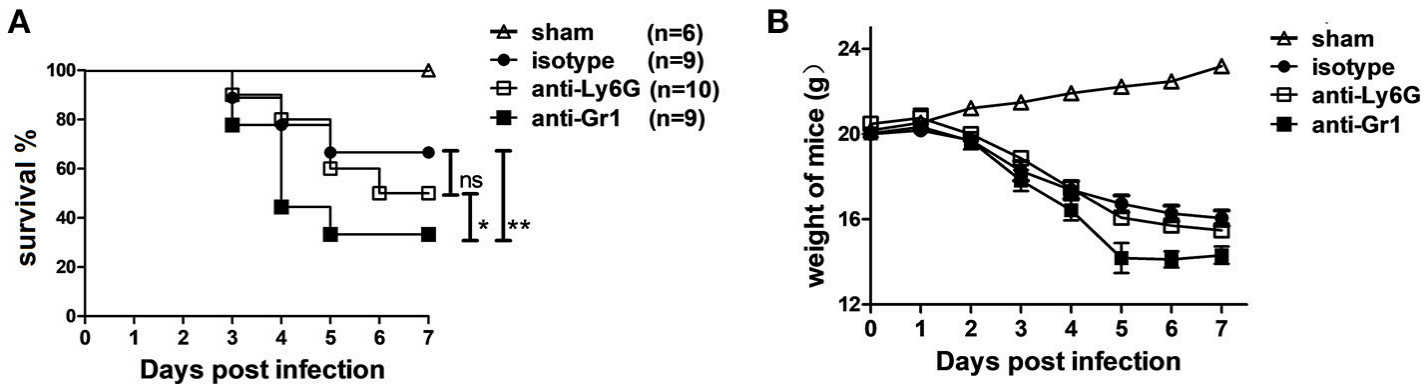
Depletion of Gr-1+ cells but not neutrophils increase susceptibility to CVB3 infection. Mice were treated with isotype, anti-Ly6G or anti-Gr-1 Abs on day −1 and 3 and subjected to 1500TCID_50_ CVB3 on day 0. The survival **(A)** and weight of mice **(B)** were monitored till 7 dpi. Sham mice with no viral infection were used as control. Results are presented as mean ± SEM; Data pooled from three independent experiments. *p < 0.05; **p < 0.01.

To examine whether neutrophils are involved in protection or pathogenesis in CVB3 infection, 1A8, RB6, or control Rat IgG2a Abs were injected into mice at 1 day before and 3 days after CVB3 infection. Throughout the 7 days infection, RB6-treated mice exhibited greater signs of sickness (ruffled fur and slight lethargy) at 3~4 dpi and died more promptly (56% dead), whereas most (80%) of isotype and IA-8-treated mice survived at this time. By 7 dpi, survival was 67 and 50% in the control and 1A-8-treated mice, respectively, whereas RB6-treated mice had decreased survival rates of 33% (*p* < 0.01, Figure 3A). The median survival of the RB6-treated mice (4.2 days, *p* < 0.05) was significantly shorter than that of the isotype- and 1A-8-treated mice (6.8 and 6.5 days, respectively). In line with that, comparable weight loss was seen in anti-Ly6G and isotype-treated mice; while Gr-1 depleted mice displayed significantly elevated weight loss (−29.2% vs. −21.5% and −18.1% of starting weight, *p* < 0.05, day 5 p.i., Figure 3B). After 5 dpi, Gr1-depleted mice showed a greater sign of recovery as compared to isotype or anti Ly6G-treated mice. Thus, susceptibility to CVB3 infection is substantially increased by Gr-1+ cells depletion, while Ly6G+ neutrophils do not much affect host anti-viral defense.

### Neutrophils are not essential but Gr-1+ cells other than neutrophils are important for limiting CVB3 replication

**Figure 4:**
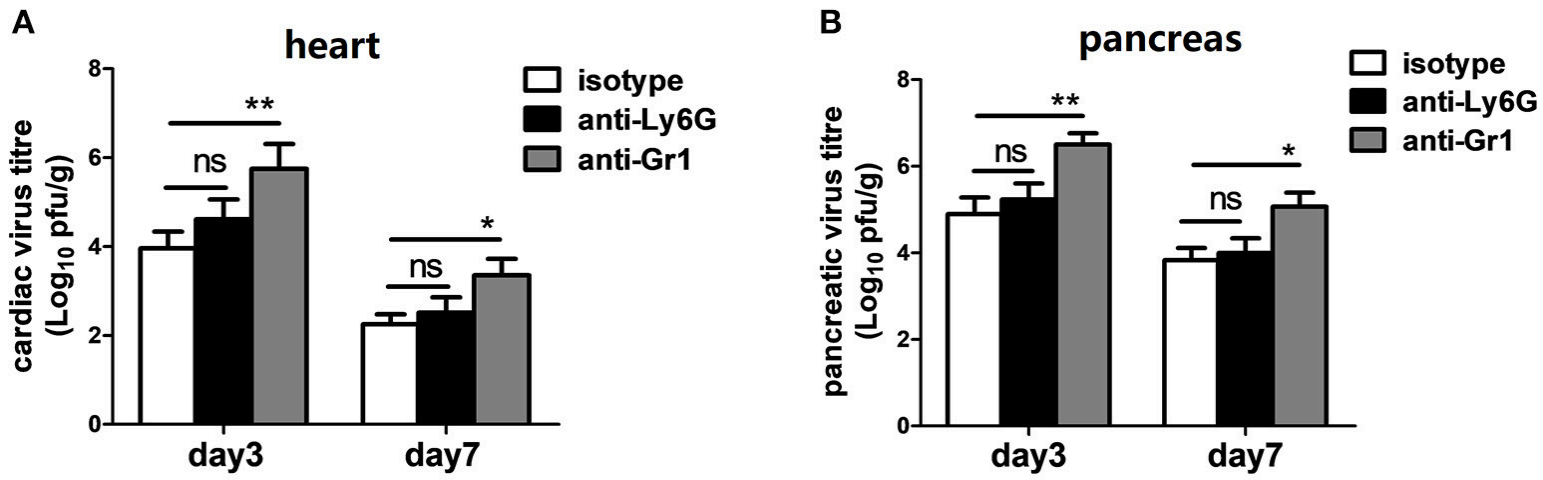
Anti-Gr-1 treated mice show an elevated cardiac and pancreatic viral burden. Mice were treated with isotype, anti-Ly6G or anti-Gr-1 Abs on day −1 and 2 and subjected to CVB3 on day 0. Viral burden in the heart **(A)** and pancreas homogenates **(B)** were assessed 3 and 7 dpi. Mice were evaluated in two independent experiments. *p < 0.05; **p < 0.01.

To test whether differences in CVB3 disease susceptibility were due to differences in viral replication, CVB3 burden in the hearts and pancreas of mice were measured. At 3 dpi, the peak of viral replication, no significant difference in cardiac viral load was observed between isotype- and anti-Ly6G-treated mice; however, depletion of Gr-1+ cells resulted in a 40-fold elevation of cardiac virus titers (5.7 vs. 3.9 Log10 pfu/g heart, *P* < 0.05) at 3 dpi compared to isotype- and anti-Ly6G-treated mice (Figure 4A). We also found an elevated effect of anti-Gr1 Ab on pancreatic viral titers (Figure 4B) at 3 or 7 dpi, indicating that neutrophil function is most likely not essential for CVB3 control in hearts and pancreas of mice. One or more types of Gr-1+ other than Ly6G+ neutrophils (Ly6C+ monocytes and macrophages or CD8+ T cells) are required to control of viral replication.

### Gr-1+ cells but not neutrophils regulate inflammatory cell infiltration and necrosis in the heart and pancreas

**Figure 5:**
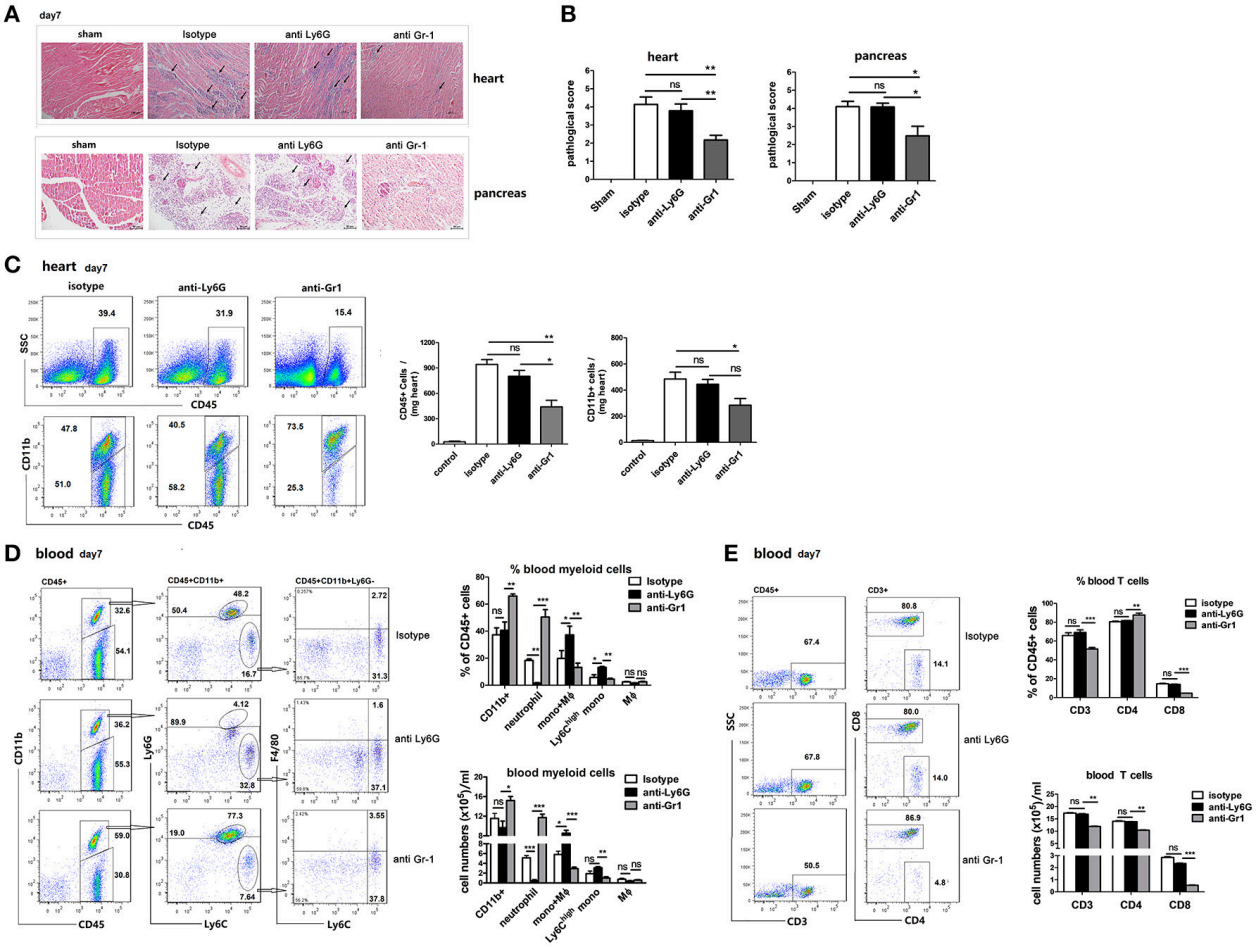
Gr-1 cell depletion ameliorate CVB3 myocarditis and pancreatitis which was associated with a reduction in blood Ly6C^high^ monocytes. **(A)** Representative image of HE-staining hearts and pancreas of CVB3-infected mice (day 7 p.i.) treated with isotype, anti-Ly6G or anti-Gr1, showing intra-cardiac immune infiltrates or damaged pancreatic acini (marked with arrows). Scale bar: 100 μm (heart) and 50 μm (pancreas). **(B)** Pathological scores of the heart and pancreas of mice are shown. Results are presented as mean ± SEM; Data pooled from three independent experiments. **(C)** Representative FACS plots of intra-cardiac total CD45^+^ leukocytes and CD11b+ cells of an individual mouse from each group (*n* = 5) on day7 p.i. Means ± SEM for frequencies and absolute numbers of cardiac CD45+ and CD11b+ myeloid cells were shown. **(D)** Representative FACS plots of CD11b+,Ly6G+Ly6C- neutrophils, Ly6C^high^ Ly6G- monocytes, and F4/80+ macrophages in the blood of CVB3-infected mice treated with anti-Ly6G or anti-Gr1 on day7 p.i. (pre-gated on CD45+ cells). Means ± SEM for frequencies and absolute numbers of neutrophils, Ly6C^high^ monocytes and macrophages within the CD45+ gate were shown. **(E)** Representative FACS plots, Means ± SEM for frequencies and absolute numbers of blood CD3+, CD4+, and CD8+ T cells were shown. Similar results were obtained in three separate experiments (*n* = 6). *p < 0.05; **p < 0.01; ***p < 0.001.

To examine whether neutrophils are involved in acute cardiac or pancreatic infiltration and disease progression, heart and pancreas sections from 1A-8-, RB6-, and isotype-treated mice that were infected with CVB3 were stained with H&E and examined by light microscopy. The severity of myocarditis in anti-Ly6G-treated mice on 7 dpi, as assessed by histology, was not significantly different from that in isotype control mice (Figure 5A). Large confluent areas of myocardial necrosis and cellular infiltration were seen in hearts of both isotype- and anti-Ly6G-treated mice. In contrast to that, only a moderate sign of cardiac inflammation was observed in anti-Gr1 Ab-treated mice at 7 dpi. (Figures 5A,B). In line with that, in hearts, total infiltrating CD45+ cells, and CD11b+myeloid cells were significantly decreased in anti-Gr1-treated mice compared to those in anti-Ly6G and isotype-treated mice (Figure 5C). Similar differences were found in the extent of pancreatic disease among different Ab-treated groups. At 7 dpi, isotype- and anti-Ly6G-treated BALB/c mice had extensive pancreatic tissue damage characterized by widespread coagulative necrosis, acinar cell lysis (marked with arrows), and inflammatory infiltration after CVB3 infection. However, pancreatic tissue from Gr1-depleted mice showed disease resolution, some of the exocrine tissues appeared normal and inflammatory infiltrate was reduced (Figure 5B). These results indicate that Ly6C^high^ monocytes and monocyte-derived macrophages, but not neutrophils, contribute to myocardial and pancreatic infiltration and tissue damage.

### Depletion of Gr-1+ cells but not neutrophils reduces Ly6C^high^ monocytes and CD8+T cells in the blood

To understand the mechanisms involved in the increased morality but reduced cardiac immune infiltration in Gr-1-depleted mice, and to determine whether depletion of circulating neutrophils affects Ly6C^high^ monocytes and T cell response, we examined the composition of blood mononuclear cells of anti-Gr1-treated mice. We isolated immune cells (CD45+) from the blood of anti-Gr1-treated mice and compared the relative proportions of neutrophils (CD11b+Ly6G+Ly6C-), Ly6C^high^ monocytes (CD11b+Ly6G-Ly6C^high^), macrophages (CD11b+F4/80+), and CD3+T cells with those in anti-Ly6G or isotype-treated mice at 7 dpi. (Figure 5D). We found that the proportion and total numbers of CD11b+ myeloid cells and CD11b-CD45+ lymphoid cells at day 7 were similar between Ly6G-depleted mice and controls, consistent to the histological analysis of the percentage of inflammation in the heart. However, anti-Gr1-treated mice contained ~25% more CD11b+ myeloid cells and ~25% less CD11b- lymphoid cells than Ly6G-depleted and control mice did. Compared to isotype Ab-treatment, anti-Ly6G treatment led to a significant decrease in neutrophil population but a significant increase in the proportion and total numbers of Ly6C^high^ monocytes as well as Ly6C^med^F4/80- monocytes in the blood (Figure 5D). While anti-Gr1 administration showed a nearly 3-fold increase in the frequency and numbers of neutrophils (18.1–50.8% within CD45+cells, *p* < 0.001) but a reduction in the proportion and numbers of circulating Ly6C^high^ monocytes (6.5–4.5% within CD45+cells, *p* < 0.05), when compared to isotype-treated mice (Figure 5D). Gr1 depletion also reduced ~15% of blood CD3+T cells, and remarkably reduced ~70% blood CD8+T cells (Figure 5E) as compared to anti-Ly6G or isotype treatment indicating a robustly enhanced viral replication in the circulation of mice after Gr1+cells depletion.

Our results suggest that following anti-Gr1 Ab treatment, peripheral Ly6C^high^ monocytes, and CD8+T cells are efficiently depleted, leading to robustly elevated viral replication which induces BM neutrophil expansion and blood perfusion. Reduced cardiac infiltration of circulating monocytes and monocyte-derived macrophages leads to reduced immune infiltration and damage in the hearts and pancreas of mice. Although anti-Ly6G Ab is meant to deplete neutrophils, it actually generates a high population of Ly6C^med^ monocyte, constituting an alternative supplements for the cardiac immune infiltrates. Thus, Gr-1+ monocytes and macrophages, not neutrophils are important in control CVB3 replication and contribute to inflammatory immune infiltration in CVB3 infected hearts and pancreas.

### Depletion of Gr-1-cells, but not neutrophils decreases cardiac pro-fibrotic TGF-β expression and fibrosis

**Figure 6:**
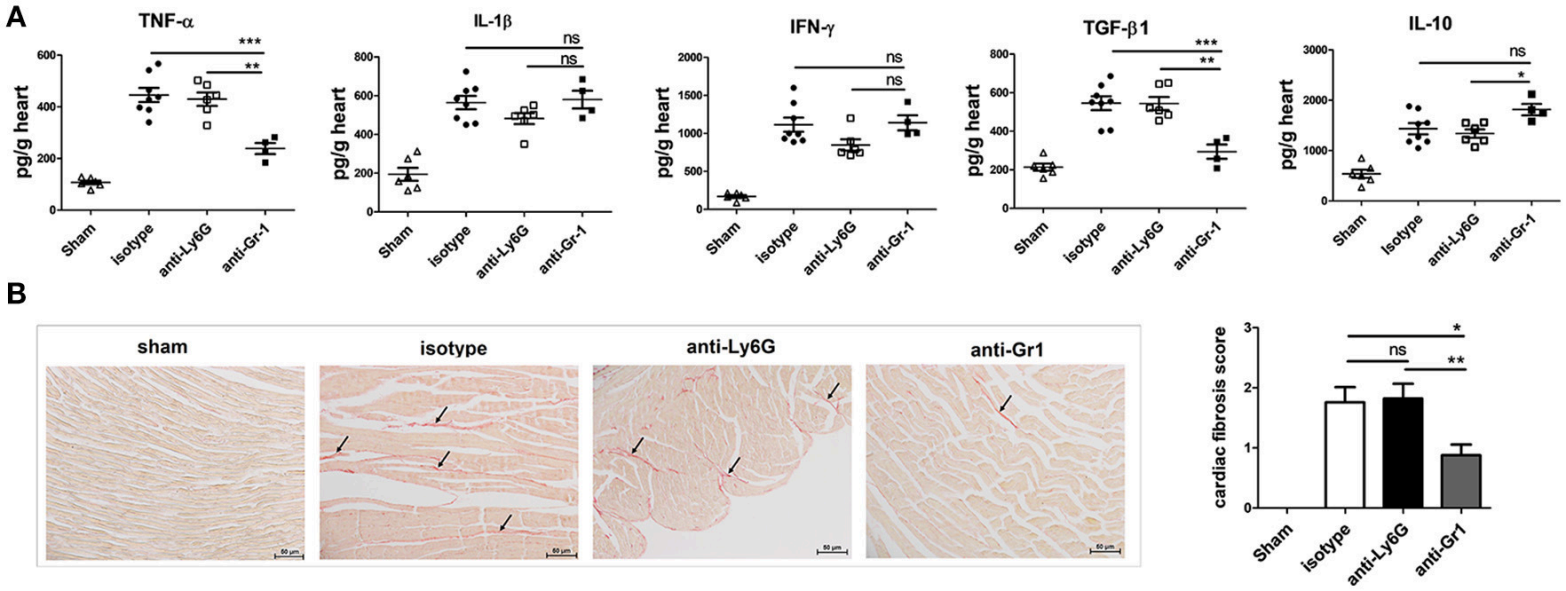
Anti-Gr1-treated mice have decreased levels of profibrotic cytokines and fibrosis in the heart. **(A)** Levels of TNF-α, IL-1β, IL-10, IFN-γ, and TGF-β from heart homogenates of anti-Gr1-, anti-Ly6G-, and isotype-treated mice at 7 dpi as determined by ELISA. Individual experiments were conducted at least three times, and data were presented as the means ± SEM of 5–8 mice per group and are normalized to wet heart weight. *p < 0.05; **p < 0.01; ***p < 0.001. **(B)** Cardiac fibrosis on day 21 after CVB3 infection was assessed as the area of the heart section with collagen deposition, stained red with sirius red (as shown by arrows). Individual experiments were conducted three times with 7 to 10 mice per group, with one representative heart with fibrotic changes shown for each group.

The local cytokine pattern is critical in determining whether fibrosis and cardiomyopathy develop, we then analyzed whether peripheral depletion of neutrophils resulted in changes in cytokine levels in the heart homogenates at 7 dpi. There were no significant differences in cardiac TNFα, IL-1β, TGFβ, and IL-10 levels (Figure 6A) between Ly6G- and isotype-treated mice. In contrast, significantly decreased cardiac levels of TNFα and TGF-β were observed following anti-Gr1 but not anti-Ly6G-treatment (Figure 6A), as compared to those seen in isotype-treated mice. Level of TGF-β, the key mediator of cardiac fibrosis, was most markedly decreased (*p* < 0.01) while level of IL-10 was significantly elevated in heart homogenates of anti-Gr1-treated mice than that in anti-Ly6G- or isotype-treated mice. No significant differences were observed in cardiac IFNγ level between anti-Gr1 and isotype treated mice (*P* > 0.05; Figure 6A). However, anti-Ly6G treatment reduced IFNγ production in the heart (*P* > 0.05) and in the blood (*P* < 0.05).

Virus-induced cardiac inflammation is prone to trigger heart pathological remodeling and tissue fibrosis. To explore the role of Gr-1+ cells on the development of cardiac fibrosis, we treated mice with anti-Gr1 or anti-Ly6G Abs on days −1, 3, 6, 9, 12, 15, and 18 dpi. Heart sections from mice were stained with sirius red to detect collagen deposition and fibrosis. Comparable levels of cardiac fibrosis were induced in isotype- and anti-Ly6G-treated mice at 21 dpi. While myocardial fibrosis was significantly decreased in mice treated with anti-Gr1 showing much less red staining for collagen deposition (Figure 6B). Thus, Gr-1+ monocytes and macrophages other than neutrophils are the driving CD11b+ subsets mediating increased cardiac pathology including severe myocarditis and cardiac fibrosis in CVB3 infection by up-regulating TNF-α and TGF-β production in the heart.

## Discussion

In this study, we try to explore that whether neutrophils or Gr-1+ cells are required to limit viral replication and modulate local inflammatory response in the progression of CVB3 myocarditis and pancreatitis. We found that upon CVB3 infection, neutrophils were the first innate immune cells recruited to the myocardium and pancreas of mice in large numbers. Circulating neutrophils alone played a dispensable role in control of virus and the progression of CVB3 disease, since peripheral absence of Ly6G+ neutrophils resulted in no change in viral clearance or the resolution of organ histopathology. In contrast, peripheral absence of Gr-1+ cells caused exacerbation of CVB3 replication and CVB3 disease (including myocarditis, pancreatitis, and cardiac fibrosis), indicating that Gr-1+ cells, but not neutrophils play a major role in determine the resistance to CVB3 infection and the pathogenesis of viral myocarditis as well as cardiac fibrosis in BALB/c mice.

Neutrophils are the first-line defense to extracellular pathogens (mostly) through production of cytokines, chemokines, and anti-microbial peptides (Nauseef and Borregaard, 2014). However, they are poorly studied with respect to viral infection. During CVB3 infection, IL-17A expression is up-regulated in the hearts of mice after CVB3 infection (Yang et al., 2011), which is thought to potentiate early neutrophil infiltration into the myocardium (Epelman et al., 2015). We confirm an early and dominant influx of neutrophils into the hearts and the pancreas of mice peaking on 3 dpi. (Figures 1C,D), preceding that of monocytes and T cells. During this antecedent infiltration, neutrophils may modulate the recruitment and activation of monocytes/macrophages and T cells by secreting various patterns of cytokines and chemokines. Therefore, we hypothesized that circulating neutrophils might be involved in host defense against CVB3 and in disease susceptibility or progression. We found that anti-Ly6G Ab, specifically targeting neutrophils, reduced circulating neutrophil numbers by over 95% at 3 dpi (Figure 2B), but hardly affected viral replication (Figure 4), cardiac immune infiltration (Figures 5A,B), and cardiac fibrosis (Figure 6), showing that these cells are not required for controlling CVB3 replication and dispensable for the cardiac or pancreatic histopathology in the early phase of viral infection. Mice depleted of Gr-1 cells exhibited a robust expansion of circulating Ly-6G+ neutrophils (Figure 2B), but were nonetheless highly susceptible to CVB3, also indicating that peripheral neutrophils are insufficient for host defense against CVB3. In accordance with our data, recent studies indicate that lung-recruited neutrophils have no effect on pneumonia virus of mice (PMV) replication or histopathological lung injury (Cortjens et al., 2016). Robust Th17-induced airway neutrophils are unable to control mouse adenovirus type 1 (MAV-1) infection or virus-induced pulmonary inflammation (McCarthy et al., 2014). Treatment of mice with 1A8 mAb does not alter virus replication or disease progression during intranasal infection of mice with HSV type 1 (HSV-1) (Wojtasiak et al., 2010). Our research extends the spectrum of infections for which neutrophils play no essential roles to include Coxsackievirus B3.

However, since neutrophils are continuously replenished from bone marrow and moving fast into the sites of infection, Ab-mediated depletion strategy is less effective at reducing numbers of BM neutrophils and cardiac-resident neutrophils. The current data may not appropriately reflect the function of BM neutrophils and cardiac-resident neutrophils. Meanwhile, our data reveal that anti-Ly6G Ab treatment seems to down-regulate Ly6G expression on membranes of pre-neutrophils instead of blocking or clearing these cells. It results in an induction of a relatively high proportion of Ly6G-F4/80-Ly6C^med^ monocyte-like population (constituted 30~40% of CD11b+ cells) in the blood at 7 dpi (Figure 5D) which may constitute an alternative supplements for the cardiac immune infiltrates. These data may account for our finding that anti-Ly6G-facilitated neutropenia leads to a comparable severe myocarditis on 7 dpi as seen in isotype-treated mice irrespective of efficient peripheral neutrophil depletion throughout the infection. Therefore, further study is needed to compare role of local neutrophils, holding unique phenotypes and functions, with peripheral patrolling uneducated neutrophils in the pathogenesis of CVB3 myocarditis.

Although Ly6C^med−high^ monocytes constitute roughly 3~10-fold less numerous than circulating neutrophils during the initial 3~5 days after CVB3 infection, CD11b^+^Ly6G^−^Ly6C^high/med^ monocyte and macrophage lineages comprise the major population of cardiac infiltrates in the later phase of CVB3 myocarditis (Fairweather et al., 2005; Huber, 2016). Reduced macrophage and T cell infiltration is associated with reduced virus elimination in acute CVB3 myocarditis (Seizer et al., 2012). IL-12/STAT4 pathway of IFN-γ production protects against CVB3 replication during acute CVB3 myocarditis via elevating cardiac macrophage populations (Fairweather et al., 2005). All these data indicate that cardiac monocytes/macrophages are important in clearing local viral burden. Gr-1+ cells comprise neutrophils, monocytes (Mo), macrophages (MΦ), DCs, and CD8+ T lymphocytes (Egan et al., 2008). By comparing data from neutrophil depletion and Gr-1 cells depletion, we can conclude that cardiac Gr-1+ Ly6C^med−high^ Mo/MΦ and CD8+T cells are essential for limiting viral replication (Figure 4) and host survival (Figure 3). Our observation is in consistent with previous study that RB6-depletion of Gr-1+ cells leads to exacerbated virus replication, disease severity and mortality during intranasal HSV-1 infection (Wojtasiak et al., 2010).

Although anti-Gr1 Ab treatment increases susceptibility of mice to CVB3 infection by promoting viral replication at 3 and 7 dpi, it effectively suppresses the severity of viral myocarditis as well pancreatitis (at 7 dpi, Figure 5A). Along with that, the absolute numbers of cardiac infiltrated CD45+ leukocytes are substantially reduced at 7 dpi (Figure 5C). Both Gr1+Ly-6C^high^ Mo/MΦ and CD8+ T lymphocytes play detrimental role in the progression of CVB3 myocarditis. Ly-6C^high^ Mo/MΦ, one of the most important pro-inflammatory myeloid cells, are vividly recruited to the heart in response to CCL2 (MCP-1) in the early phase of CVB3 infection (Shen et al., 2004) and in acute myocardial ischemic injury (Hilgendorf et al., 2014). They orchestrate cardiac inflammatory response by secretion of inflammatory cytokine. Blocking mono/MΦ intra-cardiac influx *in vivo* by targeting CCL2 exhibits reduced myocardial inflammatory injury (Yue et al., 2011). And differentially polarized macrophages shaped by the cardiac environment (including hormone-orchestrated local cytokine milieu, ER stress, NLRP3 inflammasome activation) have major roles in determining the outcome of CVB3-induced heart disease (Sanmarco et al., 2017). Apart from mono/MΦ, susceptibility to myocarditis is intensively dependent on αβT response to CVB3 infection. CD8+ T cells contribute to host susceptibility and severity of CVB3 disease (cardiac inflammation and necrosis) by influencing T and macrophage infiltration into the myocardium (Opavsky et al., 1999). CD8^+^CTL responses are reported to be correlated with myocarditis susceptibility. The autoimmune CD8^+^CTLs are the dominant pathogenic factor in CVB3-myocarditis (Liu et al., 2012). Transfer of CD8^+^CTLs into BALB/c mice is capable of inducing myocarditis in CVB3-infected and uninfected mice (Huber et al., 2002). Although CD8+ cells constitute only 10–20% of the cardiac infiltrates, they play essential role in myocarditis and cardiac damage, since in their absence NK cell and macrophage recruitment are greatly reduced (Henke et al., 1995). All the above data indicate autoimmune CD8+ CTLs are major mediators of cardiac damage during CVB3 infection. We demonstrate that anti-Gr1 Ab but not anti-Ly6G Ab treatment leads to a significantly 70% reduction in peripheral CD8+ T cell population (Figure 5E), therefore leading to reduced inflammatory and necrotic pathology in hearts of mice. Our result is in consistent with the previous report that depletion of CD8+ T cells markedly diminishes myocarditis and enhances survival, despite elevates virus titers (Henke et al., 1995).

It is of particular interest that Gr-1+ cells contribute to the fibrotic changes of the virus-infected myocardium. We demonstrate that absence of Gr-1+ cells (mostly Ly6C^high^ monocytes and macrophages) results in less progression to cardiac fibrosis on 21 dpi (Figure 6B). Virus-triggered myocarditis leading to inflammatory cardiomyopathy represents the most common cause of chronic heart failure in young patients (Gaaloul et al., 2014). Heart-infiltrating inflammatory cells include granulocytes, monocytes, T, and B cells which release various cytokines (such as IL-17, −6, −1, −10, −12, IFN-γ, TGF-β, and TNF-α), chemokines and matrix metalloproteinases. These cytokine signatures profoundly influence the heart pathological remodeling, inflammatory dilated cardiomyopathy and extracellular matrix deposition. The local secreted cytokines in the myocardium contribute great to fibrotic changes of cardiomyocytes. Among them, TGF-β is the most prominent cytokine to mediate the transition from acute inflammation to fibrosis. TGF-β stimulates non-myocyte to proliferate and secrete profibrotic molecules that expand the interstitium, increase stresses imposed on mutant myocytes, and promote myocyte death with resultant focal fibrosis in hearts (Teekakirikul et al., 2010). TGF-β signaling has been shown to mediate fibroblast differentiation of heart-infiltrating prominin-1+ progenitor cells and tissue fibrosis via Smad Proteins (Kania et al., 2009). Other than TGF-β, TNF-α is highly implicated in tissue fibrosis via synergistical interaction with TGF-β (Udalova et al., 2016). Our data reveal anti-Gr1-, but not anti-Ly6G-treatment significantly reduces the production of TGF-β and TNF-α in the hearts of mice (Figure 6A), therefore leading to less collagen deposition and reduced cardiac fibrosis on day 21 after infection. We also notice an increased cardiac IL-10 production in anti-Gr1-treated mice (Figure 6A). IL-10 is reported to suppress TGF-β-induced Smad-miRNA-21-mediated BM fibroblast progenitor cell activation and fibrotic signaling and thus modulates cardiac fibrosis (Verma et al., 2017). Thus, our data indicate that Gr-1+ cells may promote fibrogenesis and heart dysfunction in the CVB3 myocarditis model via increasing TGF-β and TNF-α-mediated inflammation while limiting IL-10 production.

Taken together, neutrophils are among the first responders to CVB3 infection in the heart and remain in large numbers throughout the development of acute myocarditis and pancreatitis. Yet peripheral neutrophils do not appear to play a major role in either host defense or tissue histopathology. Gr-1+ cells other than neutrophils are critical to limit virus replication but promote cardiac and pancreatic inflammatory injury as well as cardiac fibrosis during CVB3 infection. One mechanism by which Gr-1+ cells promote viral myocarditis and fibrosis may involve specific cytokine expression patterns. Understanding the role of neutrophils and Ly-6C^high^ Mo/MΦ during CVB3 infection may provide insight into the pathogenesis of viral myocarditis and fibrosis and the outcome of immune cell-mediated therapies.

## Author contributions

WX conceived and supervised the project, wrote the manuscript; DX, PW, and JY performed the experiments; ML and LW interpreted data and participated discussion; QQ discussion; All authors approved the final version of the paper.

### Conflict of interest statement

The authors declare that the research was conducted in the absence of any commercial or financial relationships that could be construed as a potential conflict of interest.
